# A novel homozygous *UMOD* mutation reveals gene dosage effects on uromodulin processing and urinary excretion

**DOI:** 10.1093/ndt/gfx066

**Published:** 2017-06-10

**Authors:** Noel Edwards, Eric Olinger, Jennifer Adam, Michael Kelly, Guglielmo Schiano, Simon A Ramsbottom, Richard Sandford, Olivier Devuyst, John A Sayer

**Affiliations:** 1 Institute for Cell and Molecular Biosciences, Newcastle University Medical School, Newcastle upon Tyne, UK; 2 Institute of Physiology, University of Zurich, CH-8057 Zurich, Switzerland; 3 Renal Unit, Newcastle upon Tyne Hospitals NHS Foundation Trust, Newcastle upon Tyne, UK; 4 Academic Department of Medical Genetics, Cambridge Biomedical Campus, Cambridge, UK; 5 Institute of Genetic Medicine, Newcastle University, International Centre for Life, Newcastle upon Tyne, UK

**Keywords:** gout, homozygous mutation, Tamm-Horsfall protein, tubulointerstitial kidney disease, uromodulin

## Abstract

Heterozygous mutations in *UMOD* encoding the urinary protein uromodulin are the most common genetic cause of autosomal dominant tubulointerstitial kidney disease (ADTKD). We describe the exceptional case of a patient from a consanguineous family carrying a novel homozygous *UMOD* mutation (p.C120Y) affecting a conserved cysteine residue within the EGF-like domain III of uromodulin. Comparison of heterozygote and homozygote mutation carriers revealed a gene dosage effect with unprecedented low levels of uromodulin and aberrant uromodulin fragments in the urine of the homozygote proband. Despite an amplified biological effect of the homozygote mutation, the proband did not show a strikingly more severe clinical evolution nor was the near absence of urinary uromodulin associated with urinary tract infections or kidney stones.

## INTRODUCTION

Autosomal dominant tubulointerstitial kidney disease (ADTKD) is a heterogeneous group of rare kidney diseases characterized by interstitial fibrosis with tubular atrophy causing a slowly progressive chronic kidney disease (CKD) [1]. Mutations in the *UMOD* gene coding for uromodulin are the most common genetic cause of ADTKD. Uromodulin (also known as Tamm–Horsfall protein), the most abundant protein in normal urine, is produced by the cells lining the thick ascending limb (TAL) of the loop of Henle. Recent insights point to roles for uromodulin in the regulation of salt transport in the TAL, protection against urinary tract infections (UTIs) and kidney stones and regulation of innate immunity [2]. Accurate methods to measure uromodulin in urine have recently been described [3], permitting demonstration that urinary levels of uromodulin reflect renal function and tubular activity in population cohorts [4, 5].

Rare mutations in *UMOD* are the major cause of ADTKD, a condition that leads to CKD and end-stage renal disease (ESRD) [1, 6]. More than 200 *UMOD* mutations (>95% missense, >50% targeting cysteines) have been described. These mutations lead to endoplasmic reticulum (ER) retention of mutant uromodulin in TAL cells, causing tubulointerstitial damage [7, 8]. There are no established genotype–phenotype correlations in ADTKD caused by classic *UMOD* missense mutations and large intra familial variability in presentation has been described [9]. An unusual indel mutation in *UMOD* has been associated with a particularly mild clinical evolution in several families [10]. Here we report an exceptional case of ADTKD in a consanguineous family who presented with early-onset gout and renal disease with a novel homozygous mutation in *UMOD* (p.C120Y).

## CASE REPORT

**FIGURE 1:**
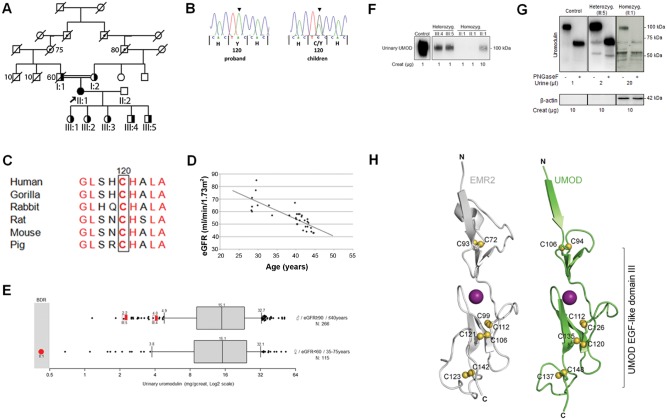
Genetic and biochemical investigation of the p.C120Y *UMOD* mutation. (**A**) Pedigree diagram with proband (II:1; arrow). Females are denoted by circles and males by squares. The homozygous individual is shaded and heterozygous individuals are half shaded. Presumed carriers of the disease allele are shown with a dotted symbol. Age of death is indicated. (**B**) Chromatograms showing the c.359G>A (p.C120Y) homozygous (proband) and heterozygous (children) missense mutation. (**C**) Conservation of the mutated amino acid C120 residue (boxed) among eutherian mammals. (**D**) Plot of age (in years) versus eGFR (Chronic Kidney Disease Epidemiology Collaboration equation) demonstrating progressive decline in renal function in the proband (index case II:1). (**E**) Urinary uromodulin levels in three individuals from the investigated family (red symbols; II:1, III:4 and III:5) compared with reference populations (black symbols; matched for gender, age and eGFR). For each proband, urinary uromodulin was measured in duplicate and the mean value plotted. Characteristics of the reference populations are shown on the right side; reference uromodulin levels are represented as box and whisker plots with whiskers representing the 10th and 90th percentiles. Urinary uromodulin is expressed in mg/g creatinine and values plotted on a log2 scale. BDR, below detection range. (**F**) Representative western blot analysis of urinary uromodulin in a control individual and two heterozygous carriers (III:4 and III:5) and the homozygous (II:1) individual from the investigated family. Two independent urine samples were loaded for the homozygous patient as well as a 10× more concentrated sample, and all the samples were run on the same blot under non-reducing conditions. The loading was normalized for urinary creatinine content as indicated. (**G**) Representative western blot analysis of *N*-deglycosylated urinary uromodulin in a control individual, a heterozygous carrier (III:5) as well as the homozygous patient (II:1). The indicated amount of urine (in µL) for each individual was reduced using DTT and loaded without or with PNGaseF treatment, as indicated. All the samples were run on the same blot. Lower panel western blot shows detection of β-actin in the homozygote urine sample only. β-actin western blot loading was normalized for urinary creatinine content as indicated and all the samples were run on the same blot. (**H**) The p.C120Y mutation resides in EGF-like domain III. On the left, the crystal structure of the EGF-like domain V of human *EMR2* (PDB code 2BOU) is shown. Stabilizing disulfide bonds are shown in yellow and the bound Ca^2+^ ion is shown as a purple sphere. On the right, a homology model of the EGF-like domain III of *UMOD* is shown. The predicted stabilizing disulfide bonds are shown in yellow and the bound Ca^2+^ ion as a purple sphere. Previously reported *UMOD*-associated disease mutations include p.C106Y (EGF-like domain II) and p.C112R, p.C126R, p.C135S, p.C135G, p.C148Y and p.C148W (all within the EGF-like III domain).

A 40-year-old Pakistani woman (index case, II:1, Figure 1A) was referred for the assessment of CKD and early-onset gout. She first showed hyperuricaemia and gout at the age of 28 years, with multiple recurrences despite allopurinol treatment. At presentation she was normotensive; her serum creatinine was 104 µmol/L (1.17 mg/dL) with an estimated glomerular filtration rate (eGFR) of 54 mL/min/1.73 m^2^. Her serum urate was elevated at 380 µmol/L (6.39 mg/dL) with a reduced fractional excretion (FE) of urate at 0.4%. Urinalysis revealed no proteinuria. There was a persistent but low (>10, <40/mm^3^) level of leucocytes in the urine on microscopy, but no haematuria. Renal ultrasound imaging showed small kidneys that were structurally normal (bipolar lengths 9.4 cm), with no cysts. A kidney biopsy was not performed. The parents were distantly consanguineous and her mother, age 75 years, is known to have CKD (eGFR 37.0 mL/min/1.73 m^2^) with gout (Table 1; Figure 1A). Her father died at 60 years of age following a cardiac event. There was no other history of renal disease or gout, despite the large pedigree (Figure 1A).

**Table 1:** Clinical, genetic and biochemical characteristics of the family members

Patient	Gender	Genotype p.C120Y	Current age (years)	Highest serum uric acid (µmol/L)	FE urate (%)	First attack of gout (years)	Current serum creatinine (µmol/L)	Current eGFR (CKD-EPI; mL/min/1.73m^2^)	ΔGFR (mL/min/1.73 m^2^/year)
Homozygote									
II:1	F	Y/Y	44	614	0.4	28	131	42.6	−3.6
Heterozygotes									
I:1	M	C/Y	Deceased age 60	NA	NA	NA	NA	NA	NA
I:2	F	C/Y	75	NA	NA	64	123	37.0	NA
III:1	F	C/Y	26	360	1%	None	81	87.0	NA
III:2	F	C/Y	24	318	1%	None	78	>90	0
III:3	F	C/Y	22	322	1%	None	80	>90	0
III:4	M	C/Y	21	377	1%	None	114	79	−3.5
III:5	M	C/Y	15	363	5%	None	82	>90	NA

CKD-EPI, Chronic Kidney Disease Epidemiology Collaboration.

The family history, suggesting autosomal dominant inheritance, and the associated early-onset gout with FE of urate prompted genetic testing for *UMOD* mutations. Sanger sequencing of the 11 exons of *UMOD* identified a single homozygous base change c.359G>A in exon 4, leading to the novel missense variant p.C120Y affecting a conserved cysteine (reference transcript NM_001008389) (Figure 1B and C). All children (III:1–5; 15–26 years of age) were heterozygous for the p.C120Y mutation (Figure 1A and B); they all exhibited a low FE of urate but none had experienced clinical gout (Table 1). Urinalysis was bland in all children and renal function was largely preserved, excepting III:4 who had a demonstrable decline in eGFR over the last 2 years. The mutation segregated from both parents (I:1 and I:2; Figure 1A), who were heterozygous for the allele. Since presentation, the proband showed slowly progressive CKD (currently stage 3b) with an eGFR falling from 53.5 to 42.6 mL/min/1.73 m^2^ over the last 3 years (−3.6 mL/min/1.73 m^2^/year; Figure 1D). Compared with her 75-year-old heterozygous mother, the proband showed a more rapid progression of CKD and earlier gout (Table 1). Specific questioning, given the genetic diagnosis, confirmed that the index patient did not have any proven UTIs, episodes of colic or renal stones. The patient admitted to polyuria (six times during the day, three times at night) but no episodes of hypotension or syncope, and no enuresis. Early-onset gout was the only notable history.

### Gene dosage effect on the quantity and quality of urinary uromodulin

Fresh urine samples from the index patient (II:1) and two of her children (III:4 and III:5) were analysed for uromodulin protein levels using a validated enzyme-linked immunosorbent assay (ELISA) method [3]. When compared with a reference population matched for gender, age and eGFR, the urinary uromodulin levels, normalized to urinary creatinine excretion, were remarkably low (Figure 1E). For the heterozygous individuals (III:4 and III:5), urinary uromodulin was consistently below the 10th percentile of urinary uromodulin values from a reference population (*N* = 266), whereas the homozygous proband (II:1) was below the detection range of the ELISA. The gene dosage effect suggested by the ELISA measurement was confirmed by western blot analysis with careful normalization to creatinine to account for urinary dilution (Figure 1F): a faint signal for uromodulin could only be observed in the homozygous (II:1) individual when loading a sample 10 times more concentrated in creatinine. The qualitative analysis of uromodulin in the urine, in control conditions and after *N*-deglycosylation (Figure 1G) revealed that the homozygous proband (II:1), like the heterozygous carrier (III:5), eliminated a mutant form of uromodulin with a molecular mass similar to the wild-type, and with normal glycosylation. The homozygous and heterozygous subjects also showed abnormal fragments of uromodulin (molecular weight <60 kDa) in the urine. These fragments were not glycosylated, as shown by the lack of a mobility shift following PNGaseF treatment. The ratio of these abnormal bands to the global signal was much higher in the homozygous proband than in the heterozygous carriers. Uromodulin fragments most likely are not explained by the presence of TAL cell debris in the urine since no β-actin is detected in the urine of the heterozygote patient. Conversely, detection of β-actin only in the urine of patient II:1 confirms cellular contaminants, probably leucocytes (see case description above) in the homozygote proband’s urine (Figure 1G). Altogether, these results show both quantitative and qualitative defects of urinary uromodulin in the patients and suggest a gene dosage effect on both aspects.

### Pathogenicity of the novel p.C120Y mutation in *UMOD*

Although the crystal structure of the polymerization domain of uromodulin has recently been reported [11], there are no available structures of the epidermal growth factor (EGF)-like domain III, wherein the novel p.C120Y mutation resides. Modelling of uromodulin based on the crystal structures of EGF-like domains with putative high structural homology predicted the formation of three stabilizing disulfide bonds between cysteine residues (C112–C126, C120–C135 and C137–C148) in EGF-like domain III (Figure 1H). This cysteine pairing pattern is characteristic of EGF-like domains [12]. Consistent modelling results were obtained using the crystal structures of EGF-like domains from EMR2 [13], fibrillin-1 [14] and Del-1 [15] (Figure 1H and Supplementary Figure S1). The *UMOD* p.C120Y mutation identified in the present study is predicted to disrupt the stabilizing interaction with C135, likely resulting in protein misfolding. This effect is therefore predicted to be pathogenic (MutationTaster: ‘disease causing’; PolyPhen-2: ‘probably damaging, score 1.00’; PROVEAN: ‘Deleterious, score −4/687’). Consistently, this mutation is not found in the ExAC database (http://exac.broadinstitute.org/), which represents >60 000 human exomes.

## DISCUSSION

Mutations in *UMOD* usually segregate as heterozygous alleles in ADTKD families, leading to an autosomal dominant pattern of tubulointerstitial kidney disease. More than 90% of reported *UMOD* mutations are missense mutations clustered in exons 3 and 4 (NM_001008389). The underlying pathology is thought to be secondary to abnormal protein folding, maturation and trafficking rather than deficient levels of uromodulin in the urine—as evidenced by the lack of progressive kidney disease in *Umod* knockout mice [2, 16]. The reduction in eGFR described in *Umod* knockout mice is most probably functional, secondary to altered sodium chloride handling and activated tubuloglomerular feedback; no morphological alterations were observed in these kidneys up to 3 years of age [17, 18]. *In vitro* studies have shown that different *UMOD* mutations lead to variable defects in the ER to Golgi trafficking. However, the underlying patterns and the phenotypic translations are currently unknown [2, 16]. The pathophysiological cascade, including partial ER retention and tubulointerstitial injury has been reproduced in mouse models and patient biopsies [7, 8].

The mutation p.C120Y found in this family has not been reported before in ADTKD. Disease-associated *UMOD* mutations affecting the cysteine at position 135 (C135), the putative disulfide bridge partner to C120, have previously been reported [19]. Uromodulin contains 48 cysteine residues, engaged in 24 disulfide bonds [20]. The importance of these bonds for the function of uromodulin is underscored by the many *UMOD* disease mutations that affect cysteine residues and by the fact that formation of the correct number of disulfide bonds within uromodulin is thought to be the rate-limiting step in the export of the precursor protein from the ER [21]. These data, together with the conservation of C120, the modelling results predicting disruption of a stabilizing interaction and the profound reduction in urinary uromodulin levels observed in carriers, strongly corroborate the pathogenic nature of the mutation.

Homozygous mutations in *UMOD* are exceedingly rare. There has only previously been one reported family with homozygous *UMOD* variants. Within this large consanguineous Spanish family, three individuals with a homozygous *UMOD* mutation (p.C255Y) were identified [22]. Compared with family members with a single heterozygous *UMOD* mutation, the three patients with homozygous changes presented with cystic kidneys, earlier onset of hyperuricaemia and a more rapid progression to ESRD. The homozygous case presented here does not show cystic change within the kidney but, as compared with the heterozygous mother, the proband exhibits earlier manifestations of gout and a more rapid decline of eGFR—consistent with reaching ESRD at the age of 50–55 years (comparison with other heterozygously affected patients is difficult due to their young age). The proband, however, does not have a strikingly severe form of disease when considering that heterozygous patients with ADTKD-*UMOD* show a median renal survival of 54 years [9]. There is no obvious reason for a particularly mild phenotype associated with the p.C120Y variant. Mutations in the EGF-like domain III are among the best studied and most prototypical mutations in *UMOD*. For instance, mutations in C148, C112, C126 and C135, the latter being predicted to pair with C120, are all associated with the classic presentation of ADTKD leading to ESRD around 40 years of age [7, 9, 19]. Additional studies are needed, including longer follow-up of the relatively young mutation carriers in this family.

The pathological basis for a gene dosage effect on the severity of ADTKD in relation to uromodulin processing and urinary excretion had not been tested thus far. Examination of kidney biopsies in mouse models and patients suggests that mutations in uromodulin are conferring a gain-of-function toxic effect to the tubulointerstitial compartment. Whether wild-type uromodulin helps in stabilizing the mutant form and, at least partially, alleviates its toxic effects or whether the toxic effect of mutated uromodulin is itself dose dependent remains an open question. On the other hand, the deletion of uromodulin in mouse does not lead to an ADTKD phenotype [17, 18].

We provide the first direct comparison of the urine levels and biochemical properties of uromodulin of heterozygote and homozygote *UMOD* mutation carriers within the same family. Compared with a cohort of individuals with a similar degree of CKD due to other causes, the levels of urinary uromodulin in the homozygote proband were undetectable using a sensitive ELISA. The urinary uromodulin levels were reduced in a gene dosage–dependent manner in the family, with levels in heterozygous patients detectable, but in the lower 10th percentile range of the normal population. Western blot analyses with non-reduced urinary samples confirmed the unprecedented low levels of uromodulin excretion in the proband, with a clear decrease of urinary uromodulin with each mutated allele. The biochemical properties of the secreted uromodulin were assessed in reducing conditions with native or *N*-deglycosylated urine samples (concentrated in the proband). Both the heterozygote and the homozygote carrier showed a major band around the physiological mass of ∼100 kDa, with a mature *N*-glycosylation pattern. However, in contrast to the control, *N*-deglycosylated uromodulin (∼70 kDa) as well as uromodulin fragments (∼50 kDa, insensitive to PNGaseF) were detected in the heterozygous and homozygous carriers. The ratio of these fragments was increased in the urine of the homozygous individual.

We can only speculate about the origins of the aberrant uromodulin bands detected in the patients. Since the accumulation of mutant uromodulin has been associated with cell death by apoptosis [23], cell debris could possibly contaminate the urine. Indeed, β-actin is detected in the urine of the homozygote proband, reflecting tubular cell debris or other cell types such as inflammatory cells (in the context of more advanced renal injury). Indeed, leucocytes were repeatedly detected in the proband’s urine. However, the aberrant bands are also detected in the heterozygote carrier in the absence of cell contamination. Furthermore, intracellular uromodulin would be detected primarily as a band of 80 kDa (ER-type glycans) that is sensitive to PNGaseF treatment [24]. Arguably the observed bands could thus reflect either immaturely secreted uromodulin bypassing ER quality control or intraluminal deglycosylation/degradation of mature uromodulin in these patients. Of note, mutant uromodulin has been detected in the urine of patients with heterozygous *UMOD* mutations, although at levels significantly lower than the wild-type uromodulin [24]. Arguably such mutant uromodulin may be more sensitive to luminal processing or degradation, explaining the greater abundance of fragments in the homozygous individual compared with the heterozygous carrier.

The lack of a wide range of clinical phenotypes in the homozygous proband is striking in the context of extremely low levels of urinary uromodulin. Based on the current understanding of uromodulin's physiological roles derived from murine KO studies and human association studies, one could expect that the lack of functional uromodulin in urine would be reflected in repeated UTIs, kidney stones and/or nephrocalcinosis, polyuria or hypotension [2]. Yet, only polyuria could be documented in this case. It should be kept in mind that the patient is not a knockout individual: although she has unprecedented low urinary levels of uromodulin, the residual, mutant uromodulin shows normal glycosylation and could thus play some physiological role. Furthermore, we should keep in mind that genome-wide association studies look for statistical associations with common variants and not for rare mutations.

Taken together, these findings demonstrate a gene dosage effect on uromodulin processing and urinary excretion, which does not associate with a striking severity of the clinical phenotype.

## MATERIALS AND METHODS

*Clinical and molecular genetics.* Clinical data were reviewed. Following informed consent, DNA was obtained from the affected patient and relatives where available. This study was approved by the Northern and Yorkshire Regional Ethics Committee. Genomic DNA was extracted from blood samples collected in ethylenediaminetetraacetic acid tubes using the QIAGEN Blood and Cell Culture DNA kit according to the manufacturer’s instructions.

*UMOD* sequencing was performed in the Cambridge Regional Genetics Centre, UK, via the UK Genetic Testing Network (http://ukgtn.nhs.uk/).

*In silico* tools MutationTaster (http://www.mutationtaster.org/), PolyPhen-2 (http://genetics.bwh.harvard.edu/pph2/) and PROVEAN (http://provean.jcvi.org/index.php) were used to determine missense mutation pathogenicity. HHPred [25] and Modeller [26] software were used to model *UMOD* based on the crystal structures of calcium-binding EGF-like domains from EMR2 (PDB code 2BOU) [13], fibrillin-1 (PDB code 2W86) [14] and Del-1 (PDB code 4D90) [15]. Homology models were visualized and figures prepared using PyMOL (http://www.pymol.org/).

*Uromodulin measurements.* The level of uromodulin was measured in spot urine samples using a validated ELISA that was performed as previously described [3, 4]. The capture antibody used was sheep anti-human uromucoid (Biodesign International, Saco, ME, USA; ref: K90071C). The primary antibody for detection was anti-human Tamm–Horsfall ascites (Clone1032A; Cedarlane, Burlington, ON, Canada). This antibody was revealed by an EIA-grade affinity purified goat anti-mouse (H + L) horseradish peroxidase conjugate (Bio-Rad, Hercules, CA, USA; ref: 172-1011). The standard protein used for calibration was human Tamm–Horsfall glycoprotein (>95%) (Millipore, Billerica, MA, USA; ref: AG733). The reference samples were obtained from the Cohort Lausannoise (CoLaus) and matched for age, gender and eGFR. The CoLaus study is a population-based study including 6000 people 35–75 years of age from the city of Lausanne, Switzerland, as previously described [4]. The uromodulin levels were normalized to urinary creatinine concentration, determined on the SYNCHRON LX UniCel DxC 800 system (Beckman Coulter, Brea, CA, USA), as previously described [4].

*Western blotting*. For semi-quantitative western blot analysis, the loading of urine was normalized to creatinine and samples were denatured by boiling but not reduced. For qualitative western blot analysis, all urine samples were denatured by boiling and reduced using dithiothreitol (DTT). When indicated, these samples were *N*-deglycosylated using PNGaseF (New England Biolabs, Ipswich, MA, USA) according to the manufacturer’s instructions. Volumes of all samples were adjusted with distilled water and mixed with Laemmli sample buffer (Bio-Rad), followed by separation on 7% SDS-PAGE gel. Proteins were then transferred onto a polyvinylidene fluoride membrane (Bio-Rad). Blocking and immunolabelling was performed using established protocols [27] with polyclonal sheep anti-uromodulin (K90071C; Meridian Life Science, Cincinnati, OH, USA) and monoclonal mouse anti β-actin (A2228; Sigma-Aldrich, St Louis, MO, USA) primary antibodies and an appropriate horseradish peroxidase–conjugated secondary antibody.

## FUNDING

J.A.S. is supported by the Kidney Research Fund and the Medical Research Council (MR/M012212/1). S.A.R. is a Kidney Research UK Post-Doctoral Fellow. O.D. is supported by grants from the European Community’s Seventh Framework Programme (305608 EURenOmics), the Swiss National Centre of Competence in Research Kidney Control of Homeostasis (NCCR Kidney.CH) programme, the Swiss National Science Foundation (31003A_169850) and the Rare Disease Initiative Zürich (Radiz), a clinical research priority program of the University of Zürich, Switzerland. E.O. is supported by the Fonds National de la Recherche Luxembourg (6903109) and the University Research Priority Programme **‘**Integrative Human Physiology, ZIHP’ of the University of Zürich.

## SUPPLEMENTARY DATA

Supplementary data are available online at http://ndt.oxfordjournals.org.

## CONFLICTS OF INTEREST STATEMENT

The authors have no conflicts of interest to declare. The results presented in this article have not been published previously in whole or part, except in abstract format.

## Supplementary Material

Supplementary Figure 1 revised
