# Supplementary material for: A novel homozygous UMOD mutation reveals gene dosage effects on uromodulin processing and urinary excretion
Source: Nephrol Dial Transplant. 2017 Jun 10;32(12):1994–9. doi: 10.1093/ndt/gfx066 (PMC5837645; doi:10.1093/ndt/gfx066)
Supplement: Supplementary Figure 1 revised [file gfx066_supplementary_figure_1_revised.docx]

**A novel homozygous *UMOD* mutation reveals gene-dosage effects on uromodulin processing and urinary excretion**

Noel Edwards, Eric Olinger, Jennifer Adam, Michael Kelly, Guglielmo Schiano, Simon A. Ramsbottom, Richard Sandford, Olivier Devuyst and John A. Sayer


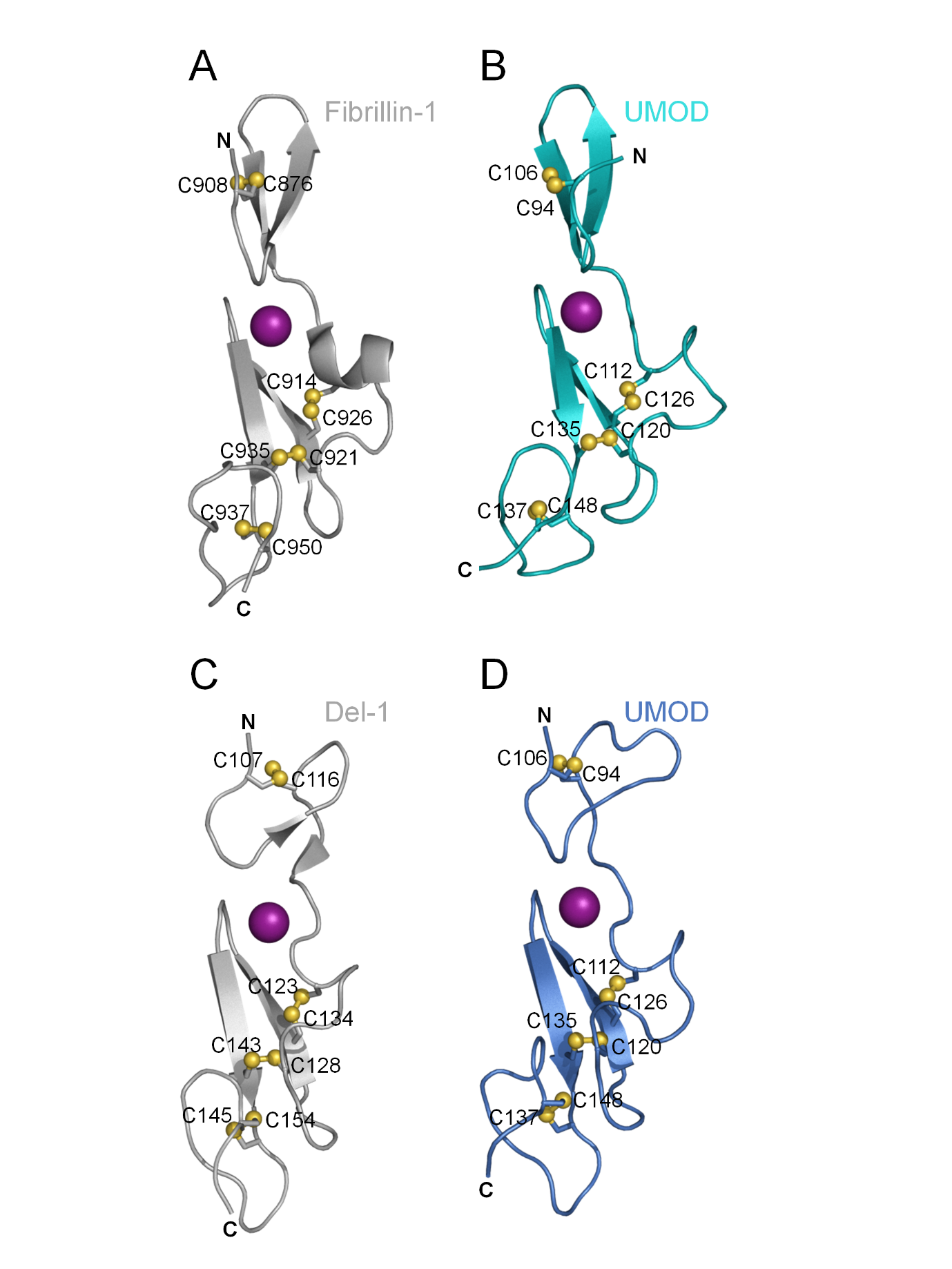
**Supplemental Figure 1. UMOD homology modelling using crystal structures of proteins containing EGF-like domains.** Crystal structure (shown in gray) and the resulting homology model of UMOD based on human fibrillin-1 (A and B, respectively) or human developmental endothelial cell locus-1 (Del-1) (C and D, respectively) consistently predicted an identical pattern of disulfide bonds (shown in yellow) within the EGF-like domain III of UMOD. Bound Ca^2+^ ions are shown as purple spheres.
